# Computerized cognitive training vs. care as usual to strengthen cognitive, motor, and (neuro)psychological outcomes in people with advanced Parkinson’s disease (TrainParC-Advanced): study protocol of a randomized controlled trial

**DOI:** 10.1186/s13063-025-09330-7

**Published:** 2025-12-24

**Authors:** Paulina M. Olgemöller, Elke Kalbe, Christina van der Linden, Michael T. Barbe, Martin Hellmich, Ann-Kristin Folkerts

**Affiliations:** 1 Medical Psychology | Neuropsychology and Gender Studies, Center for Neuropsychological Diagnostics and Intervention (CeNDI), Faculty of Medicine and University Hospital Cologne, University of Cologne, Cologne, Germany; 2 Department of Neurology, University Hospital Cologne and Faculty of Medicine, University of Cologne, Cologne, Germany; 3 Institute of Medical Statistics and Computational Biology, Faculty of Medicine and University Hospital Cologne, University of Cologne, Cologne, Germany

**Keywords:** Parkinson’s disease, Cognition, Neuropsychology, Cognitive training, Randomized controlled trial, Non-motor symptoms, Quality of life

## Abstract

**Background:**

Cognitive impairment is common in Parkinson’s disease (PD). Up to 80% of people with PD develop dementia over the disease course, heavily impacting their quality of life. While pharmacological treatment options are scarce, previous research highlights the potential benefits of non-pharmacological interventions, such as cognitive training (CT), on cognition and non-cognitive outcomes. This study’s purpose is to examine the feasibility as well as short- and long-term effects of a 5-week digital CT in people with advanced PD, compared to people with advanced PD receiving care as usual, and to explore possible predictors of CT responsiveness.

**Methods:**

This ongoing monocentric, two-armed randomized controlled trial (RCT) seeks to include 140 participants with advanced PD according to the established 5-2-1 criteria. Participants are randomized into either the experimental group, receiving a structured 5-week digital CT and additional psychoeducational videos on cognitive health, or a passive control group receiving care as usual. CT will be carried out remotely from home and supervised by the study team. Feasibility will be assessed using a training diary (e.g., motivation, satisfaction) and technical data (e.g., training duration). Neuropsychological assessments will be carried out pre- and post-CT and after a 3-month follow-up period. The primary outcome will be global cognition measured with the Montréal Cognitive Assessment (MoCA). Secondary outcomes include further cognitive, motor, and (neuro)psychological variables (e.g., quality of life, motor symptoms, mood, activities of daily living).

**Discussion:**

Studies on CT in people with PD have demonstrated positive effects on cognition, promoting the application of CT as a non-pharmacological treatment approach. However, studies targeting people in more advanced PD stages are rare or non-existent, despite it being a clinically relevant target group. Further, few studies have looked at long-term CT effects in PD, and very little data exists regarding CT predictors. Therefore, this large-cohort study of a multi-domain digital CT in people with advanced PD aims to provide insights into the effects, feasibility, and acceptability of CT within a representative sample of people with PD, allowing for statistically high-powered analyses and the identification of potential CT predictors.

**Trial registration:**

German Clinical Trials Register (DRKS) DRKS00028876. Registered on 21 November 2022

## Administrative information

Note: The numbers in curly brackets in this protocol refer to SPIRIT checklist item numbers. The order of the items has been modified to group similar items (see http://www.equator-network.org/reporting-guidelines/spirit-2013-statement-defining-standard-protocol-items-for-clinical-trials/).

**Table:** 

Title {1}	Computerized cognitive training vs. care as usual to strengthen cognitive, motor, and (neuro)psychological outcomes in people with advanced Parkinson’s disease (TrainParC-Advanced): study protocol of a randomized controlled trial
Trial registration {2a and 2b}	DRKS00028876 [Germany Clinical Trials Register (DRKS)] https://drks.de/search/en/trial/DRKS00028876 [registered November 21, 2022]
Protocol version {3}	Protocol version 6 of 31 October 2025
Funding {4}	This research is funded by STADApharm GmbH (Stadastraße 2–18, 61118 Bad Vilbel, Germany)
Author details {5a}	P. M. Olgemöller: Department of Medical Psychology | Neuropsychology & Gender Studies, Center for Neuropsychological Diagnostics and Intervention (CeNDI), Faculty of Medicine and University Hospital Cologne, University of Cologne, Germany E. Kalbe: Department of Medical Psychology | Neuropsychology & Gender Studies, Center for Neuropsychological Diagnostics and Intervention (CeNDI), Faculty of Medicine and University Hospital Cologne, University of Cologne, Germany C. van der Linden: Department of Neurology, University Hospital Cologne and Faculty of Medicine, University of Cologne, Germany M. T. Barbe: Department of Neurology, University Hospital Cologne and Faculty of Medicine, University of Cologne, Germany M. Hellmich: Institute of Medical Statistics and Computational Biology, Faculty of Medicine and University Hospital Cologne, University of Cologne, Germany A.-K. Folkerts: Department of Medical Psychology | Neuropsychology & Gender Studies, Center for Neuropsychological Diagnostics and Intervention (CeNDI), Faculty of Medicine and University Hospital Cologne, University of Cologne, Germany
Name and contact information for the trial sponsor {5b}	Faculty of Medicine, University of Cologne (Albert-Magnus-Platz, 50923 Köln)
Role of sponsor {5c}	The sponsor and the funder of this study played no role in the design of the study and collection, analysis, and interpretation of data or in writing the manuscript.

## Introduction

### Background and rationale {6a}

Parkinson’s disease (PD) is one of the most common and fastest-growing neurodegenerative diseases [1–3]. In addition to its characteristic motor symptoms (bradykinesia, rest tremor, rigidity) [4, 5], PD is characterized by several interindividual non-motor symptoms (NMS) [6, 7]. Among the most prevalent NMS in PD are cognitive dysfunctions [8–10] including mild cognitive impairment (PD-MCI) and dementia (PDD) [11, 12]. Studies indicate that approximately 30–40% of people with PD experience cognitive impairment, such as PD-MCI [13, 14], with up to 32% already showing signs at diagnosis [14, 15]. Furthermore, studies indicate that the point prevalence of PDD in people with PD is up to 30%, and that up to 80% of people with PD develop PDD over the disease course [16–18]. Notably, cognitive impairment not only impacts the quality of life (QoL) of people with PD and their relatives [19–21] but also increases mortality rates [22] and carries significant health-economic consequences [23, 24]. To date, pharmacological treatments for cognitive impairment in PD are limited, associated with side effects [25, 26] and warrant careful consideration due to the polypharmacy already associated with PD [27, 28]. Hence, alongside pharmacological approaches, considerable efforts are underway to develop non-pharmacological interventions, such as physical exercise or cognitive interventions (e.g., cognitive training [CT]) to prevent and treat cognitive dysfunction in people with PD [26].

CT refers to standardized paper-and-pencil or computerized tasks aimed at enhancing cognitive functioning by targeting single or multiple cognitive domains (e.g., memory, attention, executive function, visuocognition, and language) through structured teaching strategies or guided practice [29, 30]. Previous research has yielded promising results regarding the effectiveness of CT among people with PD with no or slight cognitive impairment. For instance, one meta-analysis demonstrated improvements in overall cognition (*g* = 0.23, 95% *CI* 0.014–0.44) and also in specific cognitive functions, i.e., in executive functions (*g* = 0.30, 95% *CI* 0.01–0.58), working memory (*g* = 0.62, 95% *CI* 0.25–0.99), and processing speed (*g* = 0.31, 95% *CI* 0.01–0.61) [29]. Another meta-analysis also found significant effects of CT on cognition, specifically for executive functions (*g* = 0.51, 95% *CI* 0.16–0.85), working memory (*g* = 0.29, 95% *CI* 0.04–0.53), and memory (*g* = 0.35, 95% *CI* 0.03–0.66) [31]. Additionally, a systematic review [32] reported on positive effects of CT not only on cognitive outcomes but also on noncognitive parameters (e.g., depressive symptoms [*d* = 0.62], activities of daily living [ADL; *d* = 1.02], anxiety [*g* = 0.50]), although findings on noncognitive aspects remain inconsistent [33–35]. However, contradictory evidence on CT effectiveness has emerged from a recent Cochrane review and meta-analysis, which found no beneficial effect of CT on cognition in people with PD-MCI or PDD [30]. The authors concluded that the limited number of included studies (*n* = 7) and the heterogeneity of research designs, PD populations, and CT implementation strategies within these studies constrain the evidence derived from this analysis. They highlighted the potential of CT interventions, noting high adherence rates (up to 92%) among individuals with PD and the limited pharmacological options for treating cognitive dysfunction, underscoring the necessity for comprehensive large-scale trials to investigate the effectiveness of CT in PD [30].

Along with the call for trials with larger samples assessing CT in PD comes a need to explore CT feasibility, acceptability, and effectiveness across all stages of PD. Given that cognitive impairment is prone to evolve throughout disease progression [13, 14, 36], it is crucial to evaluate the feasibility and effectiveness of CT in both early and advanced PD stages [12]. Achieving this entails incorporating various levels of disease severity and cognitive impairment into studies [37]. Currently, most studies predominantly assess the effects of CT in PD based on the degree of cognitive impairment; this means they focus on people with PD without cognitive impairment, with PD-MCI [32, 34, 38–40], and very rarely with PDD [30, 41]. Within these studies, CT, specifically computerized CT, seems to be feasible for and accepted by people with PD [42–44]. By focusing on cognitive metrics, however, these studies overlook inclusion criteria along other disease dimensions, such as the severity of motor symptoms. Yet, different stages of motor symptom severity pose diverse challenges for people with PD in terms of disease-related complications, medical treatment, and functional and affective impairments [45–47]. These circumstances could directly and indirectly influence CT feasibility and benefits (e.g., motivation to conduct CT, motor skills to perform CT, and responsiveness to CT).

So far, few studies on CT have included people with PD according to their motor symptom severity [29, 33]; furthermore, these studies only included participants with mild to moderate motor disease stages. Studies on more advanced motor PD stages, targeting those with PD who face a heightened susceptibility to developing cognitive impairment and dementia during disease progression [48] but do not show dementia symptoms, remain scarce. Therefore, the goal of the current study is to address these research gaps and substantially contribute to a more comprehensive care of people with PD, specifically those in more advanced stages of the disease.

Next to CT efficacy, feasibility, and acceptability, this study also seeks to provide exploratory analyses of possible predictors of CT responsiveness. Current studies indicate that cognitive baseline performance might be one of the most prominent CT predictors [38, 41, 49, 50]; other factors (e.g., sociodemographic, clinical, or (neuro)psychological) may also be relevant, but the body of literature is still inconsistent [51–53]. Determining who benefits most from CT can further help to identify an individualized, personal medicine approach that specifically targets the needs of individuals with PD, therefore complementing the previous two research goals.

### Objectives {7}

Based on a hybrid type 1 clinical trial design [54], this study has three objectives. The first is to assess the short- and long-term effects of a 5-week digital CT intervention (“HeadApp/NEUROvitalis Digital” [55]) in people with advanced PD on global cognitive functions, with the Montréal Cognitive Assessment (MoCA) [56] as the primary outcome, and other cognitive, motor, and (neuro)psychological variables as secondary outcomes (e.g., specific cognitive functions, depressive symptoms, and severity of motor symptoms). It is expected that CT will improve global cognition and other cognitive and noncognitive functions, both in the short term and over a 3-month follow-up period. The second objective is to examine the feasibility and acceptability of the CT. Specifically, it is hypothesized that the CT intervention is feasible in terms of adherence to the training schedule, completion of training days, and technical handling and is acceptable in terms of participant motivation, satisfaction, and integration of CT into daily life. Finally, the study seeks to explore and identify possible predictors of CT responsiveness, including sociodemographic factors (e.g., age, sex, education), motor (e.g., severity of motor symptoms), and cognitive and (neuro)psychological factors (e.g., cognitive baseline level, self-efficacy, and depressive symptoms).

### Trial design {8}

The study is an ongoing monocentric, single-blinded, two-armed, superiority randomized controlled trial (RCT) with one group of participants (experimental group, EG) receiving a structured digital CT over 5 weeks (four times per week for 45 min) and the other group being a passive wait-list control group (control group, CG), receiving care as usual. In addition to CT, participants in the EG receive access to complementary psychoeducational videos on cognitive health. The study follows a parallel group design. Participants are allocated in a 1:1 ratio to EG and CG. After study completion, the CG will also receive access to CT and psychoeducational videos for ethical reasons. Following good clinical practice, all data will be collected and analyzed in a standardized manner. This protocol adheres to the “Standard Protocol Items: Recommendations for Interventional Trials” (SPIRIT) guidelines.

## Methods: participants, interventions, and outcomes

### Study setting {9}

The study takes place in Germany. The primary study site is the Department of Medical Psychology | Neuropsychology and Gender Studies of the Faculty of Medicine and University Hospital Cologne of the University of Cologne in Cologne, Germany. Participants can complete the digital CT remotely from home. Neuropsychological assessments pre- and post-CT, as well as the 3-month follow-up (FU), take place at the Department of Medical Psychology | Neuropsychology and Gender Studies in Cologne, Germany, at locations specifically leased for assessments outside of Cologne, at participants’ homes, or potentially at supporting research clinics in Germany.

### Eligibility criteria {10}

To be included, participants must be diagnosed with idiopathic PD according to UK Brain Bank Criteria [57] and meet the criteria for advanced PD, defined as either meeting at least one of the established 5-2-1 criteria (five intakes of levodopa/day, 2 h of OFF time/day, and/or 1 h of troublesome dyskinesia/day) [58, 59] according to self-report or receiving pump therapy (i.e., intrajejunal levodopa pump [Duodopa, Lecigon, or subcutaneous Produodopa] or s.c. apomorphine pump [e.g., APO-go]). Participants who underwent deep brain stimulation (DBS) also have to fulfill the 5-2-1 criteria. Additionally, participants should have age-appropriate cognition/no cognitive impairment or mild cognitive impairment (PD-MCI) according to level 1 diagnosis (*MoCA* ≥ 21 points) [60] and maintain stable medication for 4-week pre-CT and through baseline and post-test. Participants, of any gender, must be 18 years of age or older, proficient in the German language, and have access to a computer, notebook, or tablet with Internet access. Exclusion criteria include a level 1 diagnosis of dementia (PDD; *MoCA* < 21 points) [60], severe depression (Beck Depression Inventory [BDI-II] ≥ 29 points) [61], epilepsy or other conditions with seizure-like manifestations and/or disorders of consciousness (ICD-10 G40), and any neurological, psychiatric, or acutely life-threatening disease that could impact cognition (according to a doctor’s letter or self-report).

### Who will take informed consent? {26a}

Written informed consent is collected at the beginning of the baseline assessment by trained research fellows. Capacity to give informed consent is assessed in accordance with Good Clinical Practice [62] and local ethical standards and follows the established principles (i.e., understanding, appreciating, reasoning, communicating) [63]. Participants receive a simple-language version of the study information and consent form prior to their baseline assessment (via e-mail/post) and again in printed form at the beginning of the baseline assessment to ensure they have sufficient time to review the material. The research fellows provide explanations as needed and answer all questions. Participants keep a copy of the consent documents and study information and are encouraged to contact the study team with any subsequent questions. Participants who lack capacity to give informed consent are not enrolled. Should a participant lose capacity during the study, procedures will follow local ethical and legal requirements. If necessary, the participant’s legal representative will be informed, and continued participation will only occur with proxy consent and ethics committee approval. Ongoing monitoring of cognitive and functional status by site investigators allows timely identification and reassessment should any concerns about consent capacity arise.

### Additional consent provisions for collection and use of participant data and biological specimens {26b}

This study does not rely on additional consent provisions.

## Interventions

### Explanation for the choice of comparators {6b}

Comparators were chosen to closely reflect the current clinical routine (see Sect. "Intervention description {11a}").

### Intervention description {11a}

#### Experimental group

Participants engage in a 5-week training schedule using the scientifically based and standardized CT “HeadApp/NEUROvitalis Digital” [55], a class 1 medical device for the prevention and therapy of cognitive dysfunctions. The CT “HeadApp/NEUROvitalis Digital” is based on the pen-and-paper NEUROvitalis program [64], an evidence-based multidomain intervention developed for cognitive rehabilitation in older adults and neurological populations (e.g., PD). The analogue NEUROvitalis program has demonstrated efficacy and high user acceptance in several RCTs, including people with PD [65–67]. The digital version used in this study preserves the structure and cognitive-domain coverage of the original program while allowing home-based, adaptive training and automated performance tracking. Participants can access CT remotely and free of charge during study participation. For each participant, CT is supervised by a single member of the study team (author P. M. O.).

**Fig. 1:**
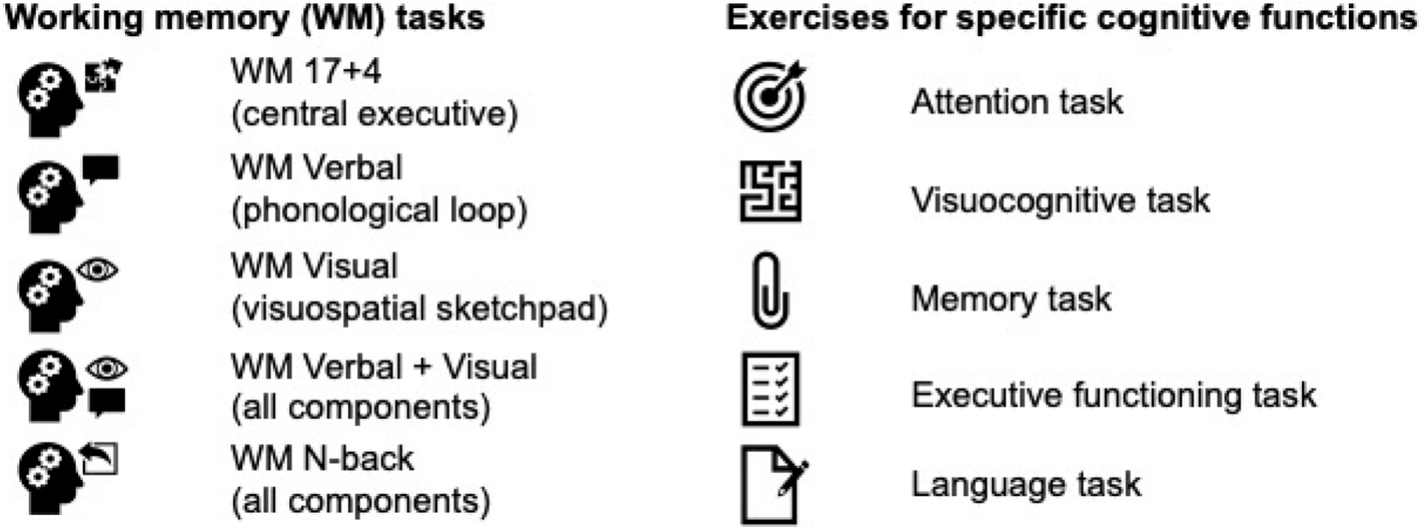
Overview of the 10 cognitive training exercises of the “HeadApp/NEUROvitalis Digital” program

Although the CT was conceptualized without specifically targeting PD, a CT schedule considering the specific cognitive profile of people with PD was developed for this study (i.e., structured rather than unstructured CT incorporating PD-relevant domains, such as working memory, memory, and executive functions [65, 66, 68]). Specifically, the structured CT schedule consists of five working memory exercises and five exercises targeting various cognitive functions: episodic memory, executive function, attention, visuocognition, and language (Fig. 1). It was specifically designed for this study and implemented into the CT program, maintaining a balanced and fixed order of exercises throughout, which participants and the study team cannot alter during the study (Table 1). The schedule consists of 20 training days to be completed over the 5-week training period. Participants are instructed to train four times per week (i.e., 4 days) for 45 min per training day, equaling 900 min in total. A training day consists of a set of three successive exercises targeting different cognitive domains (15 min each) in a prescribed order. Training frequency and duration are in accordance with previous studies on CT in PD [65, 68].

To seamlessly incorporate the CT schedule into participants’ daily lives and accommodate symptom fluctuations or other daily activities, participants can flexibly choose their training days on a day-to-day basis. Once participants access the CT website, they are led to the home screen of the CT showing the first of three exercises for the current training day. Each new day the CT website is accessed, a new training day is unlocked, and the set of three exercises for the day is accessible successively within that day. During CT, participants may take breaks within an exercise (via a pause button) and between the exercises (before starting the next exercise) or may discontinue an exercise. In case of the discontinuation of an exercise, CT automatically guides participants back to the home screen, where they can then start the next scheduled exercise. After the completion of all three exercises, a notification appears on the home screen of the CT stating that no further exercises are scheduled for this day and inviting participants to return another day.

Participants are advised by the study team to complete all three scheduled exercises for a specific training day on time, i.e., until midnight of that same day. They are also instructed that they may take breaks between exercises and return to the CT later that same day. However, in the case where not all scheduled exercises can be completed within one training day, the non-completed exercises of that training day cannot be revisited another day. Instead, the CT program automatically transitions to the next training day featuring the next set of three exercises regardless of whether all exercises of the previous training day have been completed. Therefore, once the first exercise of a training day is started, participants have until midnight to complete the scheduled three exercises; otherwise, these exercises will no longer be accessible. This process is repeated for all 20 scheduled training days.

As previous research has shown that the combination of CT with psychoeducation has proven to be particularly effective [44], in addition to CT, participants receive access to 18 psychoeducational videos (6–19 min) covering information on cognitive health and related lifestyle factors (i.e., information on physical exercise, nutrition, cognitive and social activity, and further risk and protective factors related to cognitive decline) as well as memory strategies (Table 1). The video content was developed by senior neuropsychologists and gerontologists (including authors E. K. and A. K. F.). The video production was operated by an external production company commissioned by the HeadApp/NEUROvitalis Digital company, HelferApp GmbH. For this study, video access is provided via a link sent to participants by email as the videos are currently not yet integrated into the CT program (but will be in the future). All videos can be rewatched via the link at any time for the duration of the study. Watching the videos is voluntary, but participants receive a suggestion on how to combine the CT schedule and videos. They receive a CT checklist (to be checked off by the participants) listing all 20 training days (from 1 to 20) combined with the title of 1 of the 18 psychoeducational videos (Table 1).

**Table 1:**
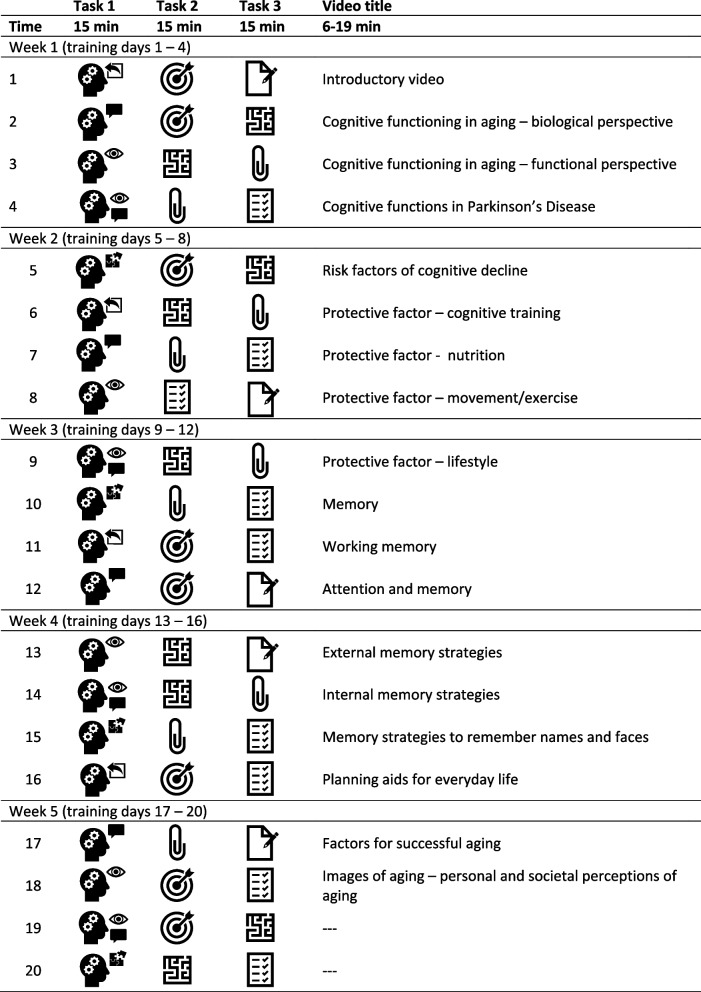
Overview of the cognitive training schedule and psychoeducational videos

The legend of the symbols for the training tasks can be derived from Fig. 1

#### Control group

To closely reflect clinical routine, participants of the CG continue their everyday life and care as usual after the baseline assessment. After a 5-week waiting period, participants complete the post-test in week 7 and the FU assessment at week 19. Following their FU assessment, participants receive 6 months of free-of-charge access to the CT program, including the CT schedule and the psychoeducational videos.

### Criteria for discontinuing or modifying allocated interventions {11b}

At any time during the study, participants may discontinue participation at their personal request without giving any reason or written statement and without incurring any disadvantage. Specific risks or harms to the participant are not expected during study participation. However, participants might experience temporary fatigue due to the length and efforts of the neuropsychological assessments or CT or may feel distressed when confronted with possible deficits during the neuropsychological assessment or CT. Reported adverse events will be recorded and analyzed, and, if necessary, the protocol will be modified accordingly. A travel and residence insurance policy has been taken out for the duration of the study. In general, study allocation will not be modified retrospectively.

### Strategies to improve adherence to interventions {11c}

To improve CT adherence among participants of the EG, various measures are implemented. First, participants of the EG receive printed information on the CT, including their individual CT access data, a step-by-step CT guide including a detailed description of the CT with images, and a brief CT diary. Additionally, participants receive a link via email to access the psychoeducational videos. To give some guidance on how to combine the CT and the videos, participants find a CT and video checklist (similar to Table 1) at the beginning of the CT diary, where they can check off each CT day and video they have completed. Secondly, participants receive printed information on the optional biweekly video consultations led by an unblinded team member (author P. M. O.), including contact details, access information, and times of the sessions. Participants can use these sessions for any CT-related queries, such as for technical problems or questions regarding certain exercises. Finally, and in addition to the previous point, a single team member (author P. M. O.) is in charge of all CT-related tasks, monitors CT progress for all participants to ensure the standardization of all procedures, and functions as the main contact person for participants. In the case that participants do not participate in the CT despite being randomized to the EG (according to their CT data), participants are contacted via telephone (and encouraged to start with the CT).

### Relevant concomitant care permitted or prohibited during the trial {11d}

All participants maintain their usual care routine without any alterations. Only the CT was added to the therapy regimen of the EG.

### Provisions for posttrial care {30}

To date, the administration of CT and neuropsychological assessment has not revealed any adverse side effects, except for occasional signs of tiredness and mild exhaustion. Therefore, no harm is anticipated from CT implementation during the study, and no posttrial care provisions are needed.

### Outcomes {12}

Based on a hybrid type 1 clinical trial design [54], our primary objective is to evaluate the efficacy of the CT. The primary outcome measure is overall cognitive function assessed by the German version of the MoCA [56, 69]. Secondary outcomes include specific cognitive functions, motor, and neuropsychiatric symptoms. Additionally, further patient-reported non-motor outcomes including QoL and ADL were defined. Non-cognitive outcome measures were selected based on recommendations of the International Parkinson and Movement Disorder Society (MDS) and the current German Society of Neurology guideline [70, 71]. CT feasibility and acceptability are also assessed as secondary outcomes. An overview of all outcome measures is shown in Table 2. A more detailed description of the outcome assessment and collection follows in Sect. “Plans for assessment and collection of outcomes {18a}”

**Table 2:**
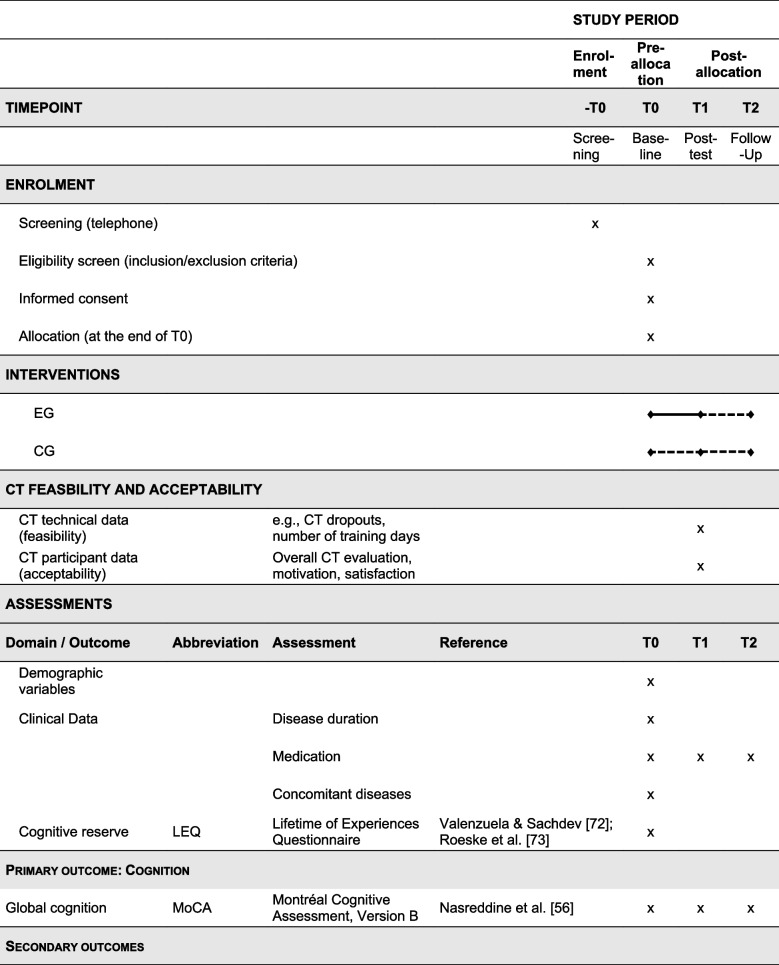

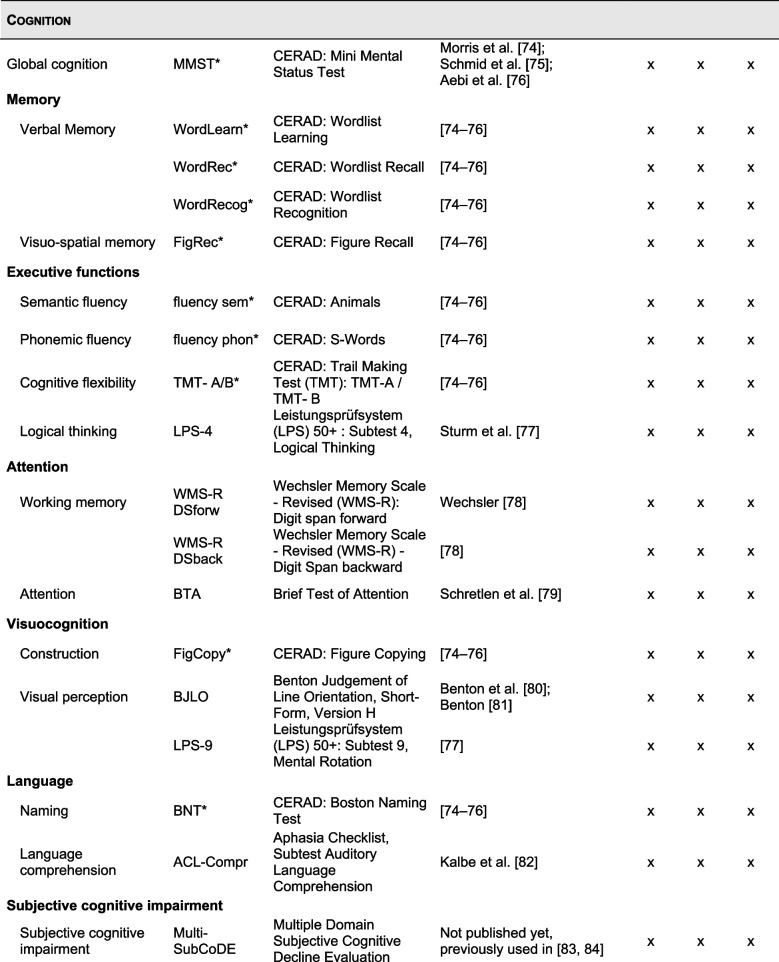

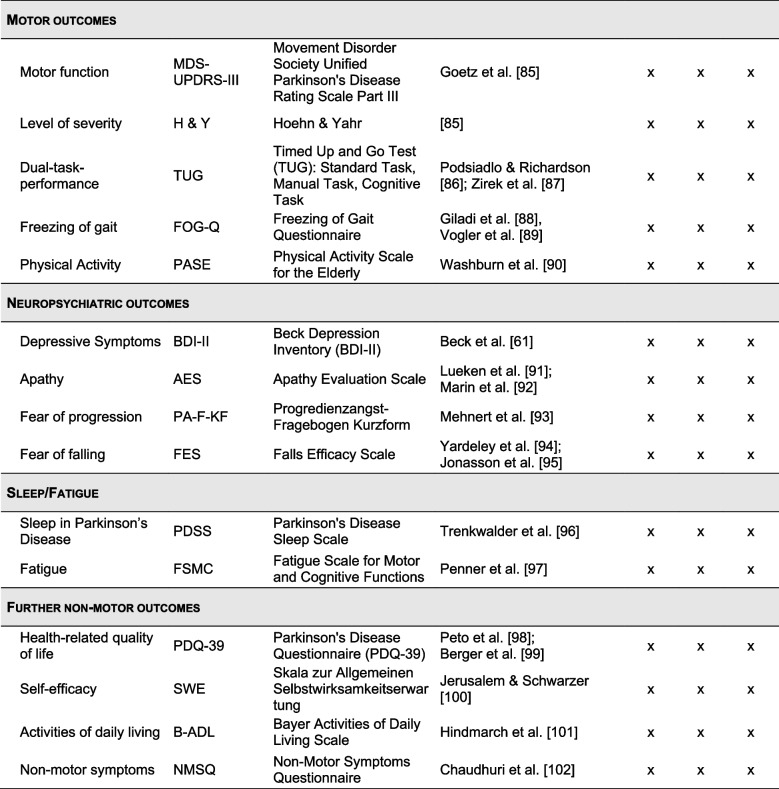
Outcomes, measurement instruments, and timepoints of assessments [56, 61, 72–102]

All items marked with a * are part of the extended version of the established neuropsychological test battery of the Consortium to Establish a Registry for Alzheimer’s Disease (CERAD-Plus) [74–76]

*Abbreviations*: *CG* control group, *CT* cognitive training, *EG* experimental group

### Participant timeline {13}

**Fig. 2:**
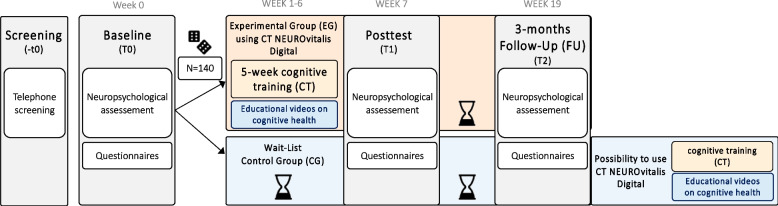
Study design

Interested individuals undergo a brief telephone screening, in which they receive information on the study purposes and an initial screening for the inclusion criteria. Those meeting the inclusion criteria are invited for baseline assessment, either at the research facility, a room leased specifically for the purpose of the assessment, their home, or a room at a supporting research facility. Figure 2 shows the study design. At baseline assessment, participants first receive detailed written information on the study and are instructed to pose any remaining questions to a trained examiner before providing written informed consent (see Sect. “Who will take informed consent? {26a}”). Following consent, all inclusion criteria are assessed in detail via a checklist. To ensure the exclusion of dementia and severe depressive symptoms, the MoCA and BDI-II are administered, respectively, and scored by the trained examiner before further assessments are undertaken. If inclusion criteria are not met, the assessment is not continued. Eligible participants continue to baseline assessment and, upon completion, are randomized to either the EG or CG (see Sect. “Sequence generation {16a}”). At the end of the baseline assessment, members of the EG receive their printed CT access data, program details (i.e., CT checklist, step-by-step CT guide, and brief CT diary), and a handout with information on the voluntary biweekly video-based consultations either in person or via post to their home by an unblinded member of the study team (author P. M. O.). Additionally, an email is sent to all participants of the EG containing a link with access to the psychoeducational videos, so they can successfully participate in the CT and watch the videos from home. In contrast, those in the CG do not receive any information on the CT but instead are instructed in person, via telephone, or via email by the same unblinded team member (author P. M. O.) to continue their care as usual until the next appointment is agreed upon. According to group allocation, a 5-week CT period (EG) or care as usual (CG) follows, concluding with a post-test at week 7 and a 3-month FU assessment at week 19. These latter assessments are again conducted at a research facility, the participants’ home, or a room leased for the occasion by a blinded examiner.

### Sample size {14}

The a-priori sample size calculation conducted in G*Power 3.1 was based on a meta-analysis [29] reporting a small efficacy of CT on overall cognitive functions. Based on this result, a small effect size is expected for global cognitive functions (*d* ≥ 0.3–0.5). With a power of 80% and a significance level of *p* = 0.05, *n* = 64 participants should be included in each of the two groups. Considering a dropout rate of 10% [38, 44], the sample calculation provided an overall sample size of *n* = 140 participants with each *n* = 70 persons randomized to either EG or CG.

### Recruitment {15}

Recruitment takes place via the Department of Medical Psychology | Neuropsychology and Gender Studies and the Department of Neurology of the University Hospital of Cologne, Germany. Information on this study is spread nationwide through brochures/flyers, posters, and announcements on the website of the University Hospital Cologne, including the website of the Department of Medical Psychology | Neuropsychology and Gender Studies. Further outreach involves engagement with Parkinson-related networks, associations, self-help groups, and former study participants associated with the Department of Medical Psychology | Neuropsychology and Gender Studies. In case these measures are not sufficient to reach the recruitment goal, various research facilities in Germany can be contacted to support the recruitment process by informing potential participants about the study (i.e., by displaying and/or handing out flyers to potentially eligible people with PD and/or referring potential study participants to the Department of Medical Psychology | Neuropsychology and Gender Studies, so they can be contacted by members of the study team).

## Assignment of interventions: allocation

### Sequence generation {16a}

The allocation sequence was computer-generated with the help of a statistician (author M. H.) using the website www.randomization.com. Allocation follows a stratified randomization procedure with permuted blocks of variable length. Participants are stratified by age (< 65 years vs. ≥ 65 years) and cognitive baseline level (*MoCA* ≤ 26 points vs. > 26 points).

### Concealment mechanism {16b}

Allocation lists are securely stored on a password-protected computer drive. Allocation is performed by one of two unblinded supervisory team members (authors E. K., A. K. F.), who both have access to the lists but are not involved in neuropsychological assessment. Group allocation is only revealed to participants at the end of their baseline assessment.

### Implementation {16c}

Blinded research fellows and/or trained medicine and psychology student assistants enroll participants and conduct neuropsychological assessments throughout the study. At the end of each baseline assessment and in the absence of the respective examiner, one research fellow (author P. M. O.) receives information on group allocation from one of the supervisory members (authors E. K., A. K. F.) and subsequently informs participants about their group allocation. From this point forward, this research fellow (author P. M. O.) is unblinded to all group allocations, and hence does not conduct any post-test or FU assessments, but handles all tasks related to group knowledge, i.e., CT set-up, monitoring of CT progress, leading the video-based consultations, overseeing study progress, and responding to inquiries to the study-specific email. All other examiners remain blinded through assessments and data management.

## Assignment of interventions: blinding

### Who will be blinded {17a}

During baseline assessments, both examiners and participants are blinded. Randomization and group allocation are carried out following baseline assessment by unblinded team members (see Sects. “Concealment mechanism {16b}” and “Implementation {16c}”). Further assessments (post-test and FU) will be carried out single-blinded, i.e., all examiners will be blind to group allocation, while participants are aware of their group. To ensure examiner blinding throughout post-test and FU assessments, participants are reminded prior to every assessment not to give hints on their group allocation. Besides blinded neuropsychological assessments, data management (e.g., data entry, data monitoring) is also carried out in a blinded manner, i.e., all paper-based and digitalized assessment data are pseudonymized and without any information on group allocation (see Sects. “Data management {19}” and “Confidentiality {27}”); therefore, all research fellows and student assistants remain blinded.

### Procedure for unblinding if needed {17b}

No unblinding procedure is planned, as participants are already aware of their group allocation. However, unblinding of the examiners may occur if participants inadvertently reveal their group allocation during neuropsychological assessment (e.g., by asking questions, talking about group allocation, or submitting the CT diary). Incidents of unintentional unblinding are documented, and further assessments are carried out by different, blinded members of the study team.

## Data collection and management

### Plans for assessment and collection of outcomes {18a}

#### Collection of outcomes

In total, participants will undergo three in-person neuropsychological assessments at baseline, post-test, and 3-month FU (Fig. 2). An overview of all assessments is presented in Table 2. All assessments are conducted in German by experienced research fellows or trained medicine or psychology student assistants. In general, demographic and clinical data (e.g., age, sex, PD duration, concomitant diseases, possible DBS surgery) as well as cognitive reserve measured by the Lifetime of Experiences Questionnaire (LEQ) [72, 73] are collected at baseline only. To control for stable medication treatment, all medication is listed and documented at the beginning of each assessment. This is followed by the neuropsychological assessment. An elaborate test battery with established psychometric instruments was set up to cover cognitive, motor, and further (neuro)psychological outcomes, which may be related to a CT effect. Regarding cognition, at least two tests per cognitive domain are used to allow a level 2 diagnosis of PD-MCI and the exclusion of PDD [103]. Additionally, participants undergo a short motor examination to assess motor outcomes (e.g., severity of motor symptoms, dual-task performance). Short breaks are administered during the assessment as needed. At the end of each neuropsychological assessment, several self-rating questionnaires are handed out to participants to fill out at home and return in a provided pre-addressed, stamped envelope. To assess CT feasibility and acceptability, participants of the EG receive a pen-and-paper CT diary at the end of their baseline neuropsychological assessment. Additionally, technical CT data will be used to assess CT feasibility. The following sections provide an overview of all outcome measures.

### Primary outcome

The primary outcome measure is the German version of the MoCA [56], a screening tool to detect MCI. The MoCA is a widely recommended screening tool for cognition in clinical trials [104]. The final score ranges from 0 to 30 points, with higher scores indicating better cognitive performance. Scores < 21 indicate PDD, scores from 21 to 25 indicate PD-MCI, and scores ≥ 26 indicate age-appropriate cognitive performance [60]. The MoCA has high sensitivity (90%) and specificity (87%) for detecting MCI [56, 60, 105] and has good reliability (Cronbach’s alpha of 0.83 [56]) and validity (receiver operating characteristics area under the curve 0.87 [106]).

### Secondary outcomes

#### Secondary cognitive outcomes

Regarding secondary cognitive outcomes, the extended version of the Consortium to Establish a Registry for Alzheimer’s Disease (CERAD-Plus) [74–76] is the basis of the neuropsychological assessments. This test battery is complemented by additional cognitive outcomes according to the LANDSCAPE study [107] to give at least two outcome measures per cognitive domain (Table 2). A second measure of global cognition is the Mini-Mental Status Test (MMST) involved in the CERAD-Plus test battery. Executive functions (i.e., semantic and phonemic fluency, cognitive flexibility, and logical thinking) are assessed using the CERAD-Plus animal and s-words, CERAD-Plus Trail Making Test (TMT) versions A and B, and the German performance test system (Leistungsprüfsystem 50+ [LPS 50+]) subtest 4 [77], respectively. Working memory and attention are assessed using the Wechsler Memory Scale—Revised (WMS-R) digit span forward and backward [78] as well as the Brief Test of Attention (BTA) [79]. Visuocognition, i.e., construction and visual perception, is measured using the CERAD-Plus figure copying, the Benton Judgement of Line Orientation (BJLO) [80, 81], and the LPS 50+ subtest 9, respectively. Verbal memory is measured using the CERAD-Plus wordlist learning, recall, and recognition subtests, while visuospatial memory is assessed using the CERAD-Plus figure recall subtest. Language, specifically naming and language comprehension, is assessed using the CERAD-Plus Boston Naming Test (BNT) and the Aphasia Checklist (ACL) auditory language comprehension subtest [82], respectively. Lastly, subjective cognitive impairment is assessed using the Multiple Domain Subjective Cognitive Decline Evaluation (Multi-SubCoDE), which has not been published but has been used previously [83, 84].

#### Secondary motor outcomes

Secondary motor outcomes include the assessment of motor function and the level of severity of motor impairment as assessed by the Movement Disorder Society’s Unified Parkinson’s Disease Rating Scale part III (MDS-UPDRS-III) [85] and Hoehn & Yahr stage [85], respectively. Assessment of the MDS-UPDRS-III is video recorded for each participant during the assessments and scored at a later time point by a blinded member of the study team. Dual-task performance is measured using the Timed Up and Go (TUG) test [86, 87] consisting of three subtests: the standard variation, the manual task, and the cognitive task. Additionally, freezing of gait and physical activity are assessed using the Freezing of Gait Questionnaire (FOG-Q) [88, 89] and the Physical Activity Scale for the Elderly (PASE) [90], respectively.

#### Secondary neuropsychiatric outcomes

Regarding neuropsychiatric outcomes, depressive symptoms are assessed according to the BDI-II [61], apathy according to the Apathy Evaluation Scale (AES) [91, 92], fear of progression according to the short version of the fear of progression questionnaire (Progredienzangst-Fragebogen Kurzform [PA-F-KF]) [93], and fear of falling according to the Falls Efficacy Scale (FES) [94, 95].

#### Secondary outcomes: sleep/fatigue

Sleep is evaluated using the Parkinson’s Disease Sleep Scale (PDSS) [96] and fatigue is assessed according to the Fatigue Scale for Motor and Cognitive Functions (FSMC) [97].

#### Secondary outcomes: further non-motor outcomes

Lastly, several other non-motor outcomes are examined, i.e., health-related QoL, self-efficacy, ADL, and non-motor symptoms, using the Parkinson’s Disease Questionnaire (PDQ-39) [98, 99], the Self-Efficacy Scale (Selbstwirksamkeitserwartung [SWE]) [100], the Bayer Activities of Daily Living Scale (B-ADL) [101] and the Non-Motor Symptoms Questionnaire (NMSQ) [102], respectively.

#### CT feasibility and acceptability outcomes

Feasibility of the digital CT is assessed in terms of participants’ ability to complete the intervention as prescribed. Indicators include CT dropout rate, number of training days completed, total duration of training, and technical handling, including any problems encountered during CT intervention. These data are collected from the CT software and via a pen-and-paper CT diary distributed to participants of the EG after their baseline assessment. Based on current literature, the CT intervention is considered feasible if CT dropouts are ≤ 14% [43] and participants completed at least 80% (i.e., ≥ 16/20) of the training days and the total training duration (i.e., ≥ 720/900 min) [30, 84]. Via optional free-text comments in the CT diary, participants can give qualitative feedback on technical handling of the CT (cf. next paragraph).

CT acceptability is evaluated based on participants’ experience with the CT. In the pen-and-paper CT diary, daily ratings capture the motivation to engage in CT on a 5-point scale (0 = “not motivated at all,” 5 = “highly motivated”) and satisfaction with each session (0 = “not good at all,” 5 = “very good”). Weekly ratings assess how well participants were able to integrate CT into their daily routines and the extent to which external factors, such as tiredness or stress, impacted training (0 = “not at all,” 5 = “very much”). At the end of the CT intervention, participants provide an overall evaluation using the German grading system (1 = “very good,” 6 = “unsatisfactory”) and indicate if they would recommend the CT (“yes”/“no”). Optional free-text comments are collected after each session, weekly, and in the final evaluation to capture qualitative feedback. The intervention is considered acceptable if the average scores across CT sessions and weekly ratings are ≥ 4/5 points and the overall evaluation reaches a score of ≥ 3.5/6 points according to the German grading system. Additionally, if more than 80% of participants recommend the CT, it will be considered acceptable. If the criteria for feasibility and acceptability are not met, the results of the CT efficacy should be interpreted cautiously as the intervention may not have been delivered effectively.

### Plans to promote participant retention and complete follow-up {18b}

Besides all measures taken to improve CT adherence for participants in the EG (see Sect. “Strategies to improve adherence to interventions {11c}”), several additional actions are taken to maintain study engagement for all participants. At baseline, all participants receive a study folder including essential information on the study (i.e., a copy of the study information, the informed consent, and insurance information). Additionally, they receive a printed handout displaying all study-relevant contact information, including the study-specific email address and phone number, which are managed by a single study team member (author P. M. O.) and are checked every weekday. Participants are actively encouraged to contact the study team via these contacts for any questions regarding the study. Next, during study participation, the progress of each participant is actively monitored and supervised by an unblinded study team member (author P. M. O.) by keeping an extensive list to monitor and document any protocol deviations, study discontinuations, or other emerging aspects (e.g., CT-relevant queries). By doing so, the study team may actively contact participants to provide support, monitor protocol deviations, and prevent dropouts. Accordingly, if participants withdraw their consent for any reason, the data of these participants will be deleted and not used for any analyses. In case of participant dropout or protocol deviations (e.g., a missing assessment), the previously collected data is still used for analyses, and measures are taken to collect as much data as possible. Following study completion, participants receive a small monetary reimbursement for each completed neuropsychological assessment (25 € per assessment, maximum total of 75 €). Upon request, participants may also receive a short neuropsychological report on the individual results across their completed neuropsychological assessments.

### Data management {19}

Data collection and management will follow the German General Data Protection Regulation. Data is only collected by members of the Department of Medical Psychology | Neuropsychology and Gender Studies. All paper-based data (e.g., informed consent, neuropsychological assessment data, scoring sheets) are stored in locked cabinets at the Department of Medical Psychology | Neuropsychology and Gender Studies in a room with restricted access. Data will be sorted by pseudonymized study ID for easy access during the duration of the study (details on the pseudonymization process are described in Sect. “Confidentiality {27}”). Data acquired on paper will be digitalized by blinded research fellows and student assistants from the study team using SPSS Statistics 29 (IBM Corp., Armonk, NY, USA). SPSS data entry and management is conducted via a dual-control principle according to pre-specified standard operating procedures and is performed continuously during study progress. Each individual step (i.e., data collection and evaluation, digitalization, and monitoring) is documented. The database is saved to a secure server of the Department of Medical Psychology | Neuropsychology and Gender Studies, accessible only through a password-protected computer to members of the study team. Backups are performed regularly. Informed consent forms are stored separately from all other paper-based data in a locked cabinet with restricted access. Digitally acquired data during assessments, i.e., MDS-UPDRS-III videos, are recorded using a standard video camera. Videos are continuously transferred to, saved, and backed up to a password-protected computer and an external encrypted hard drive with restricted access. Scoring of these data is performed by a blinded student assistant not otherwise involved in the study. The scored data is then transferred into the overall SPSS database used for this study. Digitally acquired CT data are stored via the CT program on an external server, inaccessible to members of the study team. Therefore, pseudonymized CT performance data is requested regularly from HelferApp GmbH, Gommern, Germany. Data is saved and stored in a separate data file on a password-protected computer as well as an encrypted hard drive accessible only to one member of the study team (author P.M.O) and is backed up regularly. Following good scientific practice, all data will be archived and made available upon request for at least 10 years after study completion. No data monitoring committee is used for this study.

### Confidentiality {27}

All collected data (except the informed consent) will be pseudonymized by assigning a unique, randomly generated, nonconsecutive personal identification number (study ID) to each participant. The study IDs have the following format: TPA-XXX (e.g., TPA-123). The ID numbers were generated using an online random number generator. All paper-based and digital data (i.e., neuropsychological assessment data, scoring sheets, MDS-UPDRS-III videos, and CT performance data) are encrypted and referenced using the personal study ID. Any information that might allow identification of a participant (e.g., personal information on medication plans) is made unreadable (i.e., blackened out) to ensure pseudonymization of participant data. A single list (key list) is kept for the duration of the study, linking participants’ names to their personal study ID. This key list is securely stored, saved, and regularly backed up on a password-protected computer and an external encrypted hard drive only accessible to one member of the study team (author P. M. O.). The key list will be deleted after study completion. Following deletion, all study-relevant data will be anonymous. Further information on data management and storage is described in the previous paragraph (Sect. “Data management {19}”). No participant identification details will be reported in publications.

### Plans for collection, laboratory evaluation, and storage of biological specimens for genetic or molecular analysis in this trial/future use {33}

For this study, no biological specimens are collected or analyzed. Therefore, no plans for collection, laboratory evaluation, and storage of such data are needed.

## Statistical methods

### Statistical methods for primary and secondary outcomes {20a}

The primary analysis is according to the intention to treat, with no exclusions. The change from baseline in the primary outcome MoCA is analyzed by a linear mixed model for repeated measures (MMRM) with fixed effects for baseline, group, time, and group × time interaction (ARH1-structured variance–covariance matrix over time). The primary comparison at T1 (week 9) is based on a pairwise contrast of estimated marginal means with a 95% confidence interval. Secondary outcomes (i.e., further timepoints and measures) are analyzed along the same line, however from an exploratory viewpoint only; thus, no multiplicity adjustment is required. Where available, observed changes will also be interpreted in relation to published minimally clinically important differences (MCIDs) to evaluate clinical relevance in addition to statistical significance.

### Interim analyses {21b}

No interim analysis is planned.

### Methods for additional analyses (e.g., subgroup analyses) {20b}

Predictiveness of change in the main outcome and secondary outcomes regarding baseline, age, sex, education, disease-related factors (e.g., severity of motor symptoms), and (neuro)psychological factors (e.g., cognitive baseline level, self-efficacy, and depressive symptoms) is explored by adding both main effects and interactions with group to the model equation. Moreover, models are fitted in corresponding subgroups.

### Methods in analysis to handle protocol nonadherence and any statistical methods to handle missing data {20c}

A supplementary per-protocol analysis includes only patients with three valid assessments (i.e., at T0, T1, and T2) and essential adherence to the training schedule (i.e., ≥ 16/20 (80%) completed training days in the experimental group; all control participants). The sensitivity due to missing values of the primary analysis based on MMRM is investigated by reference-based multiple imputation (e.g., jump to reference) [108].

### Plans to give access to the full protocol, participant-level data, and statistical code {31c}

Data will be available upon request. Requests for data reuse will be approved by the coordinating investigator. To gain data access, data requestors will need to sign a data access agreement.

## Oversight and monitoring

### Composition of the coordinating center and trial steering committee {5d}

This is an ongoing, monocentric study designed, executed, and coordinated at the Department of Medical Psychology | Neuropsychology and Gender Studies of the Faculty of Medicine and University Hospital Cologne of the University of Cologne in Cologne, Germany; therefore, there is no steering committee or stakeholder and public involvement group. The supervising principal investigator (author A. K. F.) and head of the department (author E. K.) provide day-to-day support for the trial and meet weekly with the research fellow coordinating and executing the study (author P. M. O.). Trained research fellows as well as medicine and psychology student assistants from the department support data collection and data management.

### Composition of the data monitoring committee and its role and reporting structure {21a}

This study does not use a data monitoring committee. However, data entry and data checks are performed via the dual control principle by blinded members of the study team. Possible conflicts or uncertainties regarding data management or data monitoring are elaborated and discussed with the supervisory members of the study team (authors E. K. and A. K. F.).

### Adverse event reporting and harms {22}

So far, to the authors’ knowledge, no adverse events related to CT conduct have been reported in the literature. Therefore, adverse events related to CT conduct are not expected for this study. In the case that an adverse event occurs that is directly connected to CT participation, the events will be documented.

### Frequency and plans for auditing trial conduct {23}

Auditing is conducted on a weekly basis for internal reasons; a quarterly report is sent to the funder.

### Plans for communicating important protocol amendments to relevant parties (e.g., trial participants and ethical committees) {25}

Important modifications to the study protocol (e.g., changes to eligibility criteria, outcomes, or analyses) will be communicated to all relevant parties in a timely manner.

## Dissemination plans {31a}

Study results will be presented at national and international conferences and published in peer-reviewed scientific journals and will, therefore, be available to all relevant parties. Additionally, it is planned to publish the results of this study in a patient-oriented manner (e.g., in a nationwide self-help group journal).

## Discussion

This ongoing two-armed RCT examines the feasibility and effects of a 5-week digital CT in people with advanced PD, compared to those with PD receiving care as usual, while also exploring potential predictors of CT responsiveness. The body of literature assessing the effects of CT in PD is expanding, with preliminary evidence of positive effects [29, 32–34]; however, these findings are primarily based on heterogeneous studies with small sample sizes (*n* ≤ 76), including participants that are typically defined along cognitive inclusion criteria, therefore limiting the generalizability of these studies.

To address these challenges and to contribute significantly to this field, the current study incorporates several important aspects. First, to our knowledge, this study represents the first large-cohort investigation of both short- and long-term effects of a digital CT in people with advanced PD. Currently, no disease-modifying therapies or medications exist that may reduce the risk of cognitive decline or dementia for people with PD [45]. Moreover, managing PD in more advanced disease stages is complex, requiring careful consideration and a comprehensive approach that integrates both pharmacological and non-pharmacological treatments [45, 46]. Digital CT, with its potential to be widely accessible and its ability to be safely integrated into existing treatment regimens, holds promise as a long-term strategy to enhance cognitive function and potentially mitigate or delay cognitive decline [45, 109]. The large sample size of this study provides a robust foundation for statistically high-powered analyses, contributing valuable insights into the effects of CT for advanced PD. Additionally, the 3-month follow-up period will enable the exploration of sustained cognitive benefits and possible delayed effects, providing data on the potential for lasting cognitive improvement in this population.

Secondly, we evaluate the feasibility and acceptability of digital CT in advanced PD, thus examining whether digital CT interventions are suitable for more advanced PD stages in terms of technical handling as well as participants’ motivation and satisfaction with CT. Given that previous studies have shown high adherence to CT among people with PD [30], such interventions — particularly digital ones — have the potential to serve as a user-friendly complementary treatment option for individuals experiencing cognitive decline or those who wish to take preventive measures against it [37]. As there is currently no widespread availability of CT within the German healthcare system, digital CT, which can be performed at home, presents even greater opportunities for individuals with PD across various disease stages. If CT proves feasible and acceptable, this study offers evidence to support the integration of digital CT interventions into the daily lives of people with PD, especially for those in more advanced disease stages who may be limited in their mobility or those living in more rural areas, where it can be difficult to receive CT outside their homes. Integrating interventions like digital CT into their daily routines could enhance people with PD’s self-management skills by enabling them to actively participate in managing their condition [110, 111].

Third, this study seeks to identify possible predictors of CT responsiveness on an exploratory basis, with the goal of determining which individuals are most likely to benefit from CT. The existing literature on predictors of CT responsiveness is highly heterogeneous. Some studies suggest that cognitive baseline levels may predict CT responsiveness [38, 44, 49, 50], while other factors (e.g., age, education, motor symptom severity, and intelligence) have also been explored, with inconclusive results [38, 44, 51–53]. The present study may therefore provide deeper insights into the predictors of CT responsiveness, potentially facilitating the development of personalized CT interventions tailored to the specific needs of people with PD. Additionally, exploring these predictors may enhance our understanding of the mechanisms underlying CT effectiveness in PD, leading to more efficient resource allocation and targeted interventions across different stages of the disease.

Fourth, in contrast to previous studies on CT in PD, which primarily focused on participants' cognitive status [40, 65], this study adopts a more clinical perspective by recruiting participants based on disease severity. This methodological shift better reflects the realities of clinical practice, where people with PD are often categorized by their overall disease progression (e.g., severity of motor symptoms) rather than isolated cognitive metrics [112, 113]. Moreover, the use of a passive CG aligns with current clinical conditions, where the majority of individuals with PD do not receive CT unless they actively pursue it. By selecting a clinically relevant sample of individuals with PD at high risk for cognitive decline, this study may provide insights that could enhance comprehensive care strategies, particularly for individuals in more advanced stages of PD who are frequently underrepresented in existing research. Addressing this gap is essential for developing interventions that are applicable, easy to administer, and beneficial in real-world clinical settings.

Despite the abovementioned strengths of this study, certain challenges may arise during the execution of the study. One of the biggest challenges may be the recruitment of 140 individuals with advanced PD. To address potential difficulties in recruitment, a comprehensive recruitment plan using a phased approach was developed by the study team. The initial phase involves distributing flyers and handouts at the University Hospital Cologne, as well as reaching out to people with PD who have previously participated in studies at the primary study site and agreed to be contacted again for further studies. Additionally, the study is promoted on the website of the University Hospital Cologne and through repeated brief calls for participants at the “Parkinson’s Day for people with PD” event at the University Hospital Cologne. In the second phase, regional and nationwide self-help groups are contacted. If desired, members of the study team may give a short presentation (either digitally or in person) on the study and its background to these groups. In the third phase, the study team will reach out to supporting research institutes and PD clinics nationwide to assist with participant recruitment (e.g., by distributing flyers or promoting the study to potential participants). Taken together, this recruitment plan opens various communication channels that can facilitate recruitment and help achieve the goal of 140 participants.

A second challenge may be the risk of missing data due to the long battery of tests and the fact that this is administered by various members of the study team. To mitigate the potential for data loss caused by inadequate data collection or management (e.g., incorrect administration of tests during neuropsychological assessments or unusable data), all members of the study team receive comprehensive training in data collection and management, supported by standard operating procedures developed for and tailored to this study; all members will be supervised by the executing study team member (author P. M. O.) as well as the supervisory study team members (authors E. K. and A. K. F.). In cases where complete study adherence is not possible, the study team tries to collect as much data as possible from the respective participant.

While the study has many strengths in place for the prevention or handling of potential challenges, certain limitations must still be acknowledged. First, since the study involves digital CT, participants must have access to a digital device with a reliable Internet connection and must be capable of using such a device to complete CT. This requirement may exclude some individuals with advanced PD, despite their eligibility based on disease stage. However, an advantage of the digital CT format is that it allows participation from individuals across Germany, including those who are immobile or unable to travel to the research facility. Second, the advanced PD stage may introduce additional challenges. These include disease-related complications, complex medical treatments, fluctuations of motor and non-motor symptoms—potentially affecting the ability to complete CT—and other complications that could impact the study’s progress or lead to a higher-than-expected dropout rate. Moreover, the complexity of care of people with advanced PD may lead to a higher rate of protocol deviations, which must be accounted for. Third, while this study aims to examine a participant pool that more accurately reflects clinical practice, it still excludes individuals with PD who are already suffering from dementia, thus precluding any conclusions on CT in people with PDD. Future research will need to build on this study’s findings to include and investigate individuals with PDD.

Fourth, since participants receive CT together with psychoeducational videos, any observed CT effects must be interpreted as resulting from the combined intervention, as the current study design does not permit separating the effects of CT from those of the psychoeducational component. Further studies using more elaborate statistical designs are needed to isolate specific contributions of each component.

In conclusion, the current study is, to the best of our knowledge, the first large-cohort study examining the effects of CT on people with PD in more advanced disease stages, thus offering class I evidence on the efficacy of digital CT in PD. CT could be an easy-to-administer, far-reaching, and safe intervention to strengthen cognitive functions in this patient group and prevent cognitive decline.

## Trial status

Protocol version 6.0, 26 November 2025. The start of the study was April 1, 2022. The trial reached first-patient-in on November 30th, 2022. Recruitment is expected to be completed by the end of 2025.

## Data Availability

Upon study completion, the final datasets used and/or analyzed during the current study will be available from the corresponding author on reasonable request. In this case, a data-sharing agreement must be signed.

## Abbreviations

ACL: Aphasia Checklist
ADL: Activities of daily living
AES: Apathy Evaluation Scale
B-ADL: Bayer Activities of Daily Living Scale
BDI-II: Beck Depression Inventory
BJLO: Benton Judgement of Line Orientation
BNT: Boston Naming Test
BTA: Brief Test of Attention
CERAD-Plus: Consortium to Establish a Registry for Alzheimer’s Disease (extended version)
CG: Control group
CT: Cognitive training
DBS: Deep brain stimulation
EG: Experimental group
FES: Falls Efficacy Scale
FOG-Q: Freezing of Gait Questionnaire
FSMC: Fatigue Scale for Motor and Cognitive Functions
FU: Follow-up
H&Y: Hoehn & Yahr
LEQ: Lifetime of Experiences Questionnaire
LPS: Leistungsprüfsystem
MCI: Mild cognitive impairment
MCID: Minimally clinically important difference
MDS-UPDRS-III: Movement Disorder Society Unified Parkinson’s Disease Rating Scale Part III
MMST: Mini-mental status test
MoCA: Montréal Cognitive Assessment
Multi-SubCoDE: Multiple Domain Subjective Cognitive Decline Evaluation
NMS: Non-motor symptoms
NMSQ: Non-motor symptoms questionnaire
PA-F-KF: Progredienzangst-Fragebogen Kurzform
PASE: Physical Activity Scale for the Elderly
PD: Parkinson’s disease
PD-MCI: Mild cognitive impairment in Parkinson’s disease
PDD: Parkinson’s disease dementia
PDQ-39: Parkinson’s Disease Questionnaire
PDSS: Parkinson’s Disease Sleep Scale
QoL: Quality of life
RCT: Randomized controlled trial
SPIRIT: Standard Protocol Items: Recommendations for Interventional Trials
SWE: Skala zur Allgemeinen Selbstwirksamkeitserwartung
TMT: Trail Making Test
TUG: Timed Up and Go Test
WM: Working memory
WMS-R: Wechsler Memory Scale—Revised
